# Cobalt Protoporphyrin Induces HO-1 Expression Mediated Partially by FOXO1 and Reduces Mitochondria-Derived Reactive Oxygen Species Production

**DOI:** 10.1371/journal.pone.0080521

**Published:** 2013-11-08

**Authors:** Xiaojun Liu, Ying Cui, Meixia Li, Haifeng Xu, Jin Zuo, Fude Fang, Yongsheng Chang

**Affiliations:** 1 The National Laboratory of Medical Molecular Biology, Institute of Basic Medical Sciences, Chinese Academy of Medical Sciences and Peking Union Medical College, Beijing, China; 2 State Key Laboratory of Brain and Cognitive Science, Institute of Biophysics, Chinese Academy of Sciences, Beijing, China; 3 Key Laboratory of Carcinogenesis and Translational Research (Ministry of Education), Department of Interventional Therapy, Peking University Cancer Hospital & Institute, Beijing, China; National Institutes of Health, United States of America

## Abstract

**Background:**

Reactive oxygen species arise in the mitochondria as byproducts of respiration and oxidase activity and have important roles in many physiological and pathophysiological conditions. The level of reactive oxygen species is regulated by a number of enzymes and physiological antioxidants, including HO-1, Sod2, catalase and COX-2, etc. And HO-1 against oxidative stress requires an increase in stress-responsive genes, such as Sod2 and catalase. Especially for the activity of HO-1, cobalt protoporphyrin is known to be a potent and effective inducer in many tissues. The transcription factor, FOXO1 is resistant to oxidative stress through downregulating reactive oxygen species production. Previous study showed that FOXO1 induces HO-1 expression by binding to HO-1 promoter. The question whether cobalt protoporphyrin induces HO-1 expression mediated by FOXO1 and subsequently lessens reactive oxygen species production remains to be elucidated.

**Results:**

Cobalt protoporphyrin enhances the expression of FOXO1 and facilitates FOXO1 binding to HO-1 promoter and increasing its transcriptional activity without influencing the FOXO1 protein stability. CoPP induces HO-1 and other oxidative stress-responsive genes expression, such as catalase, cytochrome c, Sod2, and COX-2, and decreases mitochondria-derived reactive oxygen species production, which are mediated partially by FOXO1.

**Conclusions:**

Cobalt protoporphyrin induces HO-1 and other oxidative stress-responsive genes expression mediated partially by FOXO1, and has an important role in reducing cellular reactive oxygen species level. Cobalt protoporphyrin may be a more promising therapeutic agent to upregulate some antioxidantive genes.

## Introduction

Reactive oxygen species (ROS), such as the superoxide radical, the hydroxyl radical, and hydrogen peroxide, are continuously produced in most cells under physiological conditions. Aerobic cells produce a series of ROS in normal intracellular metabolism and by external stimuli, such as inflammatory cytokines, growth factors, environmental toxins, chemotherapeutics, UV light, or ionizing radiation [1]. In pathophysiological conditions, ROS can damage proteins, lipids, and DNA, leading to cell death. Furthermore, many human diseases, including cancer, aging, diabetes, and neurodegenerative diseases, are related to mitochondrial dysfunctions provoked by ROS. Although ROS are produced in multiple cell compartments, the majority of cellular ROS (approximately 90%) contribute to mitochondrial energy metabolism. The level of ROS is regulated by a number of oxidative stress-responsive genes, such as superoxide dismutase (Sod), catalase, ATP synthase and glutathione peroxidase (Gpx). And vice versa, excessive ROS induces expression of some oxidative stress-responsive genes, such as cytochrome c (Cyt c) and cyclooxygenase-2 (COX-2). 

Heme oxygenase (HO) is the rate-limiting enzyme for breaking down heme into carbon monoxide, biliverdin, and free iron [2]. Three HO isozymes including HO-1, HO-2, and HO-3 have been identified, among which HO-1 is an inducible enzyme that induces cellular protection in the event of injury, inflammation, oxidative stress, etc, and HO-2 and HO-3 are constitutive ones. HO-1 has been proved to have several biological effects including anti-inflammatory, antiapoptotic and antiproliferative actions [3–5]. Disruption of HO-1 by siRNA attenuated the IL-19-induced reduction in ROS concentration and indicated that the IL-19-driven decrease in ROS is mediated by HO-1 expression [6]. On the other side, HO-1 was tightly regulated by ROS levels within cells [7]. The upregulation of the HO-1 gene is not dependent on some classic stress pathways or kinase cascades, but dependent on several heme-responsive elements in the 5′-UTR of HO-1. It is well known that many stressful stimuli can increase the expression of HO-1 including heme or certain other metalloporphyrins, particularly cobalt protoporphyrin (CoPP) [8]. The primary mechanism for upregulation of the HO-1 gene is through enhancing transcription of the gene [9]. CoPP is known to be a potent and effective inducer of HO-1 activity in many tissues [10–12]. Previous study indicated that CoPP-induced upregulation of HO-1 gene expression was mediated by the transcription factors Bach1 and Nrf2 in human hepatoma cells and that the underlying mechanism was attributed to the posttranscriptional destabilization of the Bach1 protein and stabilization of the Nrf2 protein in response to CoPP [13].

FOXO proteins are a group of the Forkhead family of transcription factors recognized by a conserved DNA-binding domain termed as Forkhead box or FOX. This conserved family consists of four members, FOXO1 (also known as FKHR), FOXO3 (also known as FKHRL1), FOXO4 (also known as AFX1) and FOXO6, and is a subclass of the Forkhead family of transcription factors [14]. In the absence of any cellular stimulus, FOXOs are localized in the nucleus, where they regulate transcription of their target genes. Upon activation of protein kinase B (PKB) by growth or survival factors, FOXOs are phosphorylated at their highly conserved residues (corresponding to Thr-24, Ser-256 and Ser-319 in human FOXO1), relocalize from the nucleus to the cytosol, and no longer function as transcriptional activators [15]. FOXO proteins participate in many important functions such as the regulation of cell apoptosis, cell-cycle progression, cell differentiation and metabolism, and resistance to oxidative stress [16,17]. FOXO1 involves in mTORC2 controlling the innate inflammatory response [18]. In adipocytes, free fatty acids (FFA) decreases FOXO1 protein levels in a dose-dependent manner and the FOXO1 downregulation correlated with an increase in the production of reactive oxygen species [19]. 

Previous study showed that FOXO1 induced HO-1 expression by binding to HO-1 promoter [20]. In this study, we present that CoPP increases the expression of FOXO1, furthermore upregulates HO-1 and other oxidative stress-responsive genes expression and decreases mitochondria-derived ROS production, which are mediated partially by FOXO1.

## Materials and Methods

### Chemicals and Antibodies

Cobalt protoporphyrin (CoPP) and cycloheximide are purchased from Sigma-Aldrich. Anti-FOXO1 antibody is ordered from Abcam, anti-HO1 antibody is obtained from Assay Designs, anti-Sod2 antibody is from Abclonal technology, anti-catalase antibody and anti-COX-2 antibody are from Bioworld Technology and anti-GAPDH antibody is from CW Biotechnology. All restriction enzymes are purchased from Promega, and Taq polymerase from Takara Inc. 

### Plasmids

The mouse HO-1 reporter gene construct (-1500bp-+50bp) is created by cloning the mouse HO-1 promoter fragment into the promoterless luciferase reporter gene vector pGL3 basic (Promega). The amplified construct is verified by restriction digestion and sequencing prior to its use. 

### Transfections and Luciferase Assays

HepG2 cells are grown at 37 °C, 5% CO_2_ in Dulbecco’s modified Eagle’s medium (DMEM) supplemented with 10% fetal bovine serum, penicillin (100 units/ml) and streptomycin sulfate (100 μg/ml). Cell transfection is performed with cells plated on 24-well plates according to the manufacturer’s protocol (Vigorous). Indicated amount of each expression construct is co-transfected with a luciferase reporter plasmid (200–300 ng) and the internal control plasmid pRL-TK (20–50 ng). Total amount of DNA in each transfection is adjusted to appropriate concentration per well for pcDNA3.1 (vector DNA). After incubation of transfectants for 24–48 hrs, the activity is measured by means of commercially-available kits (Promega). All luciferase assays are performed for 3 times at least.

### Isolation and culture of mouse primary hepatocytes

Primary mouse hepatocytes are isolated from 8-week-old male C57BL/6J mice and cultured in RPMI 1640 medium with 10% FBS as described previously [21]. The mouse experiment was approved by the Animal Research Committee at the Institute of Laboratory Animals, Chinese Academy of Medical Science and Peking Union Medical College.

### RNA Interference and Adenovirus Infection

Short-hairpin RNA (shRNA)-encoding DNA sequences are synthesized by Invitrogen (Carlsbad, CA) and constructed into adenovirus plasmids, and adenoviruses are prepared according to previously described procedures [22]. Adenovirus encoding FOXO1 shRNA has been described [23], shRNA against FOXO1 (shFOXO1) is 5’- GAGCGTGCCC TACTTCAAG -3’, and the sequence of control shRNA against luciferase (shControl) is 5’- CTTACGCTGAGTACTTCGA-3’. 

### RNA Isolation and Quantitative RT-PCR

Total RNA is extracted from the HepG2 cells or primary hepatocytes using Trizol-based method (Roche). About 2 μg of total RNA is reverse-transcribed into first-strand cDNA pool using SuperScript TM reverse transcriptase and random primers (Abcam). Quantitative real-time reverse-transcriptase PCR (qRT-PCR) is performed using the SYBR Green I Q-PCR kit (Promega) on a Bio-Rad CFX system. All gene expression data are normalized to β-actin or GAPDH expression levels. All primer sequences are available upon request. Specific primers are shown in Table 1.

**Table 1:** The qPCR primers details.

Primer	forward (5’-3’)	reverse (5’-3’)
β-actin	CCAGCCTTCCTTCTTGGGTAT	TGCTGGAAGGTGGACAGTGAG
HO-1	TCCAAGCCGA GAATGCTGAG	CTCCTCAGGGA AGTAGAGTG
FOXO1	GTGAACACCATGCCTCACAC	CACAGTCCAAGCGCTCAATA
Cyt C	GTTGATCTGCAAATTAAAATGCT	CACGATCTGTGGTTGTTTTAAT
Catalase	CAGCGACCAG ATGAAGCAGT	AGTGTGCCAT CTCGTCAGTG
Sod2	AGAAGTACCA CGAGGCTCTG	GATAGCCTCC AGCAACTCTC
Gpx1	CTCGGTTTCCCGTGCAATC	CTCACCATTCACTTCGCA
Atp5C	TCAAGTCTGTTATCTCCTAC	GAGGTTGGCCAGATTGTAC
COX-1	CAGAGTCATG AGTCGAAGGA	CTGGTTCTGG CACGGATAGT
COX-2	AAAACCGTGGGGAATGTATGAGC	GATGGGTGAAGTGCTGGGCAAAG

### Western Blotting

Protein is extracted from frozen organ samples or cultured hepatocytes in cell lysis buffer, and 40-60 µg of protein are loaded onto a 10% SDS-polyacrylamide gel and separated proteins are transferred to PVDF membranes. Western blot assays are performed using antibodies specific for HO-1, FOXO1, Catalase, COX-2, Sod2 or GAPDH.

### ChIP assays

The ChIP assays are performed as described previously [24]. Primary hepatocytes are treated with CoPP or DMSO, respectively. Cross-linked chromatin fragments are precipitated with control IgG or anti-FOXO1 antibody for subsequent PCR analysis. The amplification primers of mHO-1 and control mGAPDH promoter are TGTGCCATCACTACCCAGAA, AGCAGGGTAAGGCTTGGAAT and AGAGAGGGAGGAGGGGAAATG, AACAGGGAGGAGCAGAGAGCAC. 

### Measurement of mitochondrial ROS accumulation

Mitochondrial ROS accumulation are measured as previous reported [25]. In brief, intracellular production of mitochondrial ROS is measured using MitoSOX Red (Molecular Probes). For the assay, primary hepatocytes are plated in six-well plates. Cells are infected by shFOXO1 virus or control virus. After 24h, cells are exposed to 10 μM CoPP or DMSO for 18h. And cells are trypsinized and harvested, then are loaded with MitoSOX Red for 10 minutes in the dark at 37°C. Next, cells are washed twice with PBS and fluorescence is measured using flow cytometry (excitation at 510 nm, emission at 580 nm). Data analysis is performed with c-flow software and the mean fluorescence intensity is used to quantify the responses. A minimum of 10,000 cells is acquired for each sample.

### Statistical analysis

All the data are indicated as mean ± s.d. Differences among means are analyzed by Independent-sample T test and one-way analysis of variance (ANOVA) (SPSS). P value < 0.05 is considered to be significant.

## Results

### FOXO1 increases the expression of HO-1 dependence on FOXO1 binding to the promoter of HO-1

**Figure 1:**
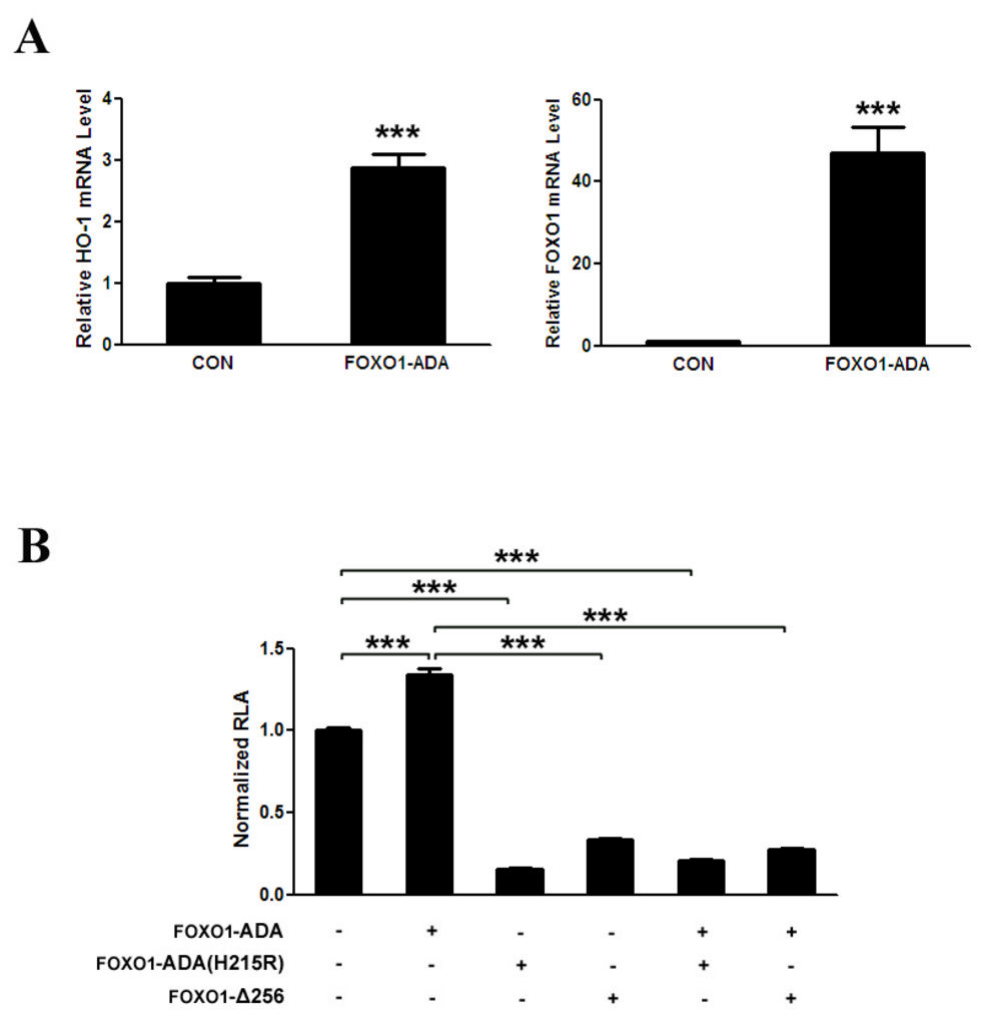
FOXO1 increases the expression of HO-1 dependence on FOXO1 combine to the promoter of HO-1. (A) FOXO1 upregulates HO-1 mRNA in HepG2 cells. HepG2 cells are transfected for 48 h with pcDNA3-FOXO1-ADA and control plasmids, after which cells are harvested, total RNA assays are carried out. HO-1 and FOXO1 mRNA are quantified by qRT-polymerase chain reaction and relative amounts of HO-1 and FOXO1 mRNA are normalized to β-actin mRNA. (B) FOXO1 enhances HO-1 promoter transcription activity dependent on FOXO1 binding to the promoter of HO-1. HepG2 cells are co-transfected with pGL3-Basic-HO-1 promoter (200 ng) and the equivalent amounts of pcDNA3-FOXO1-ADA or/and pcDNA3-FOXO1-ADA (H215R) (pCMV5-FOXO1-Δ256 or control plasmids) as indicated. *** p < 0.001.

We firstly examine whether HO-1 mRNA levels are upregulated by FOXO1. As previous study indicated [20], FOXO1 overexpression induces HO-1 mRNA level in HepG2 cells (Figure 1 A). Previous study showed that FOXO1 directly regulates the expression of HO-1 by combining with the promoter of HO-1. We also investigate the effects of different forms of FOXO1 on the promoter of HO-1 by dual luciferase reporter gene assay. FOXO1-ADA, a phosphorylation mutant that is resistant to growth factor-induced phosphorylation and inactivation by nuclear exclusion [15], increases the transcriptional activity of HO-1 promoter. To examine if the promoter activation is dependent on FOXO1 binding to the DNA, we analyze the effects of FOXO1-Δ256, a truncated FOXO1 mutant lacking the transactivation domain acts as a dominant negative inhibitor of FOXO1-mediated transcription [26], and FOXO1-ADA(H215R), which is defective in its interaction with the DNA [27,28], on the transcriptional activity of HO-1 promoter. FOXO1-Δ256 evidently decreases the basal and FOXO1-ADA-induced transcriptional activity of HO-1 promoter (Figure 1B). Interestingly, FOXO1-ADA(H215R) could not increase the transcriptional activity of HO-1 promoter, but obviously decreases the basal and FOXO1-ADA-induced transcriptional activity of HO-1 promoter (Figure 1B). These data suggest that the up-modulation of HO-1 by FOXO1 requires its physical binding with the DNA and FOXO1 mediates the effects of some collaborative factors on HO-1 promoter activation.

### CoPP increases the expression of FOXO1 and promotes FOXO1 binding to the promoter of HO-1

**Figure 2:**
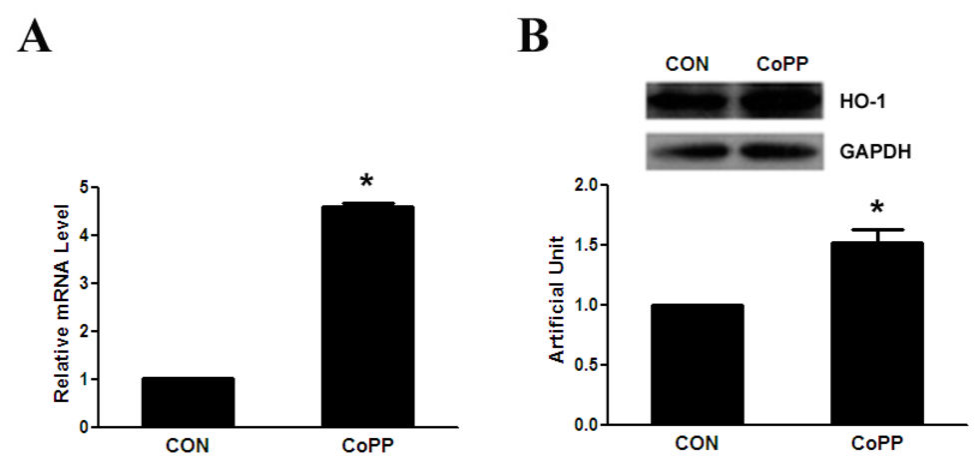
CoPP upregulates HO-1 gene expression in mouse primary hepatocytes. Primary hepatocytes are treated for 16 h with and without 10µM of CoPP, after which cells are harvested, total RNA and protein assays are carried out. (A) HO-1 mRNA is quantified by qRT-polymerase chain reaction and relative amounts of HO-1 mRNA are normalized to β-actin mRNA. (B) Protein levels are quantified by western blot as described in Methods and relative amounts of HO-1 protein are normalized to GAPDH. *p < 0.05.

**Figure 3:**
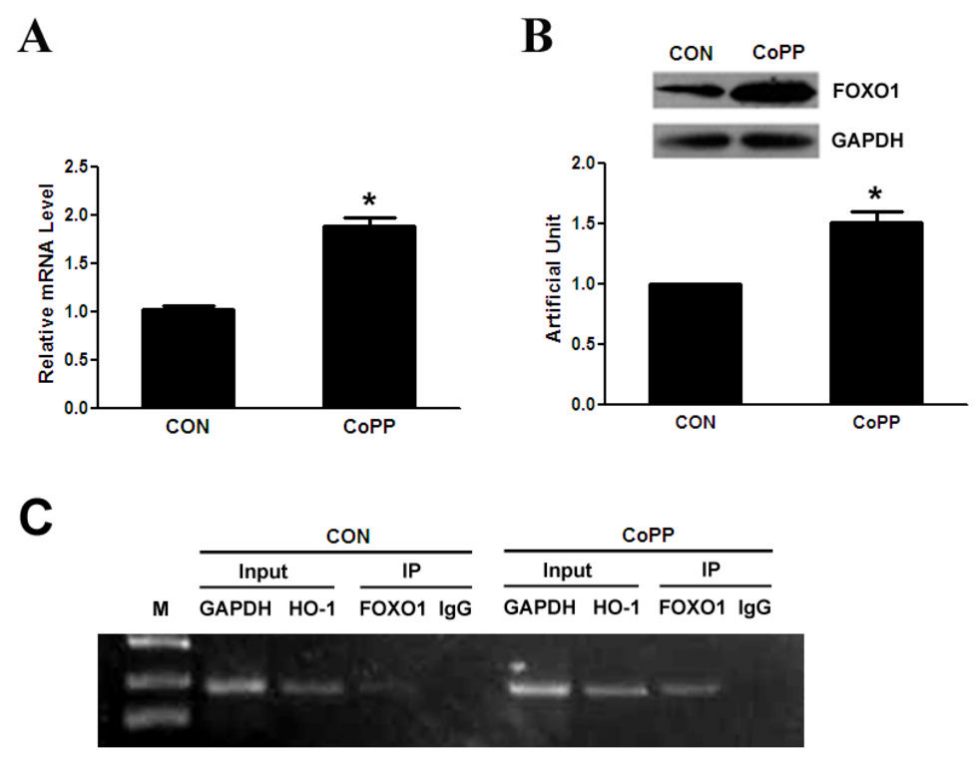
CoPP increases the expression of FOXO1 and promotes FOXO1 binding to the promoter of HO-1. Primary hepatocytes are treated for 16 h with and without 10µM of CoPP, after which cells are harvested. (A) HO-1 mRNA is quantified by qRT-polymerase chain reaction and relative amounts of FOXO1 mRNA are normalized to β-actin mRNA. (B) Protein levels are quantified by western blot as described in Methods and relative amounts of FOXO1 protein is normalized to GAPDH. (C) ChIP assay. Protein-DNA complexes are immunoprecipitated with anti-FOXO1 antibody or IgG. HO-1 promoter region harboring the FOXO1 recognition motif and control mGAPDH promoter are amplified by PCR. *p < 0.05.

It is well known that CoPP is a potent and effective inducer of HO-1 activity in many tissues [10–12]. As described in previous studies, 10 μM CoPP treatment for 24 h increases the expression of HO-1 mRNA and protein in primary hepatocytes (Figure 2A and 2B). Since FOXO1 enhanced the expression of HO-1, we examine the effect of CoPP on FOXO1 expression. Our results indicate that 10 μM CoPP treatment for 24 h increases FOXO1 mRNA and protein levels in primary hepatocytes (Figure 3A and 3B). In addition, we also investigate the effect of CoPP treatment on the ability of FOXO1 binding to the promoter of HO-1 by ChIP as described in Materials and Methods. Compared to control, CoPP treatment for 24 h increases the occupancy of FOXO1 on the promoter of HO-1 in primary hepatocytes (Figure 3C). 

### CoPP has no influence on FOXO1 protein stability

**Figure 4:**
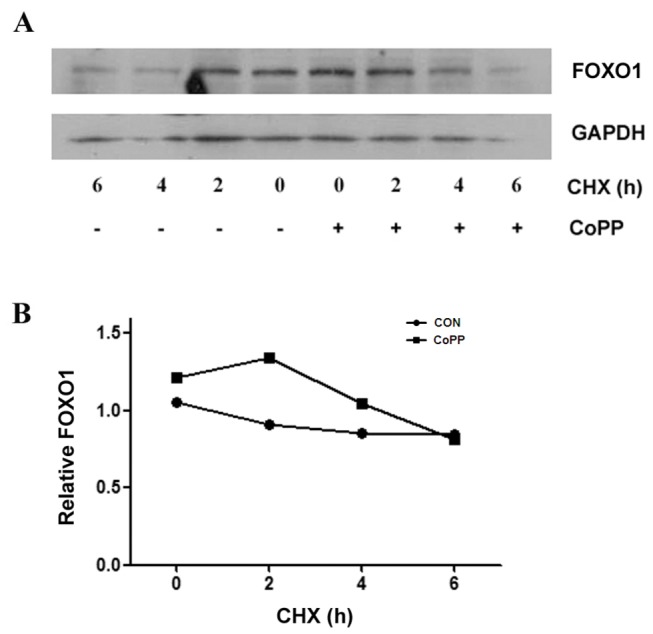
CoPP has no influence on FOXO1 protein stability. Primary hepatocytes are treated with or without 10 µM CoPP for 16 h, followed by treatment with 100 µg/ml cycloheximide (CHX) for the indicated times, after which cells are harvested and total protein is isolated as described in Materials and Methods. Proteins are separated by 10% SDS-polyacrylamide gel, transferred to a PVDF membrane, and probed with anti-mouse FOXO1 and GAPDH specific antibodies. (A) FOXO1 protein levels with or without CoPP. (B)The relative amounts of FOXO1 proteins are normalized to GAPDH.

Previous study indicated that CoPP increases HO-1 expression by posttranscriptional destabilization of the Bach1 protein and stabilization of the Nrf2 protein [13], we ask whether CoPP affects FOXO1 protein stability. Our results manifest that FOXO1 protein in cells treated with CoPP is not resistant to cycloheximide (CHX), an inhibitor of protein synthesis and indicate that CoPP has no influence on FOXO1 protein stability (Figure 4A and 4B).

### CoPP induced-expression of HO-1 and other oxidative stress-responsive genes are mediated partially by FOXO1

**Figure 5:**
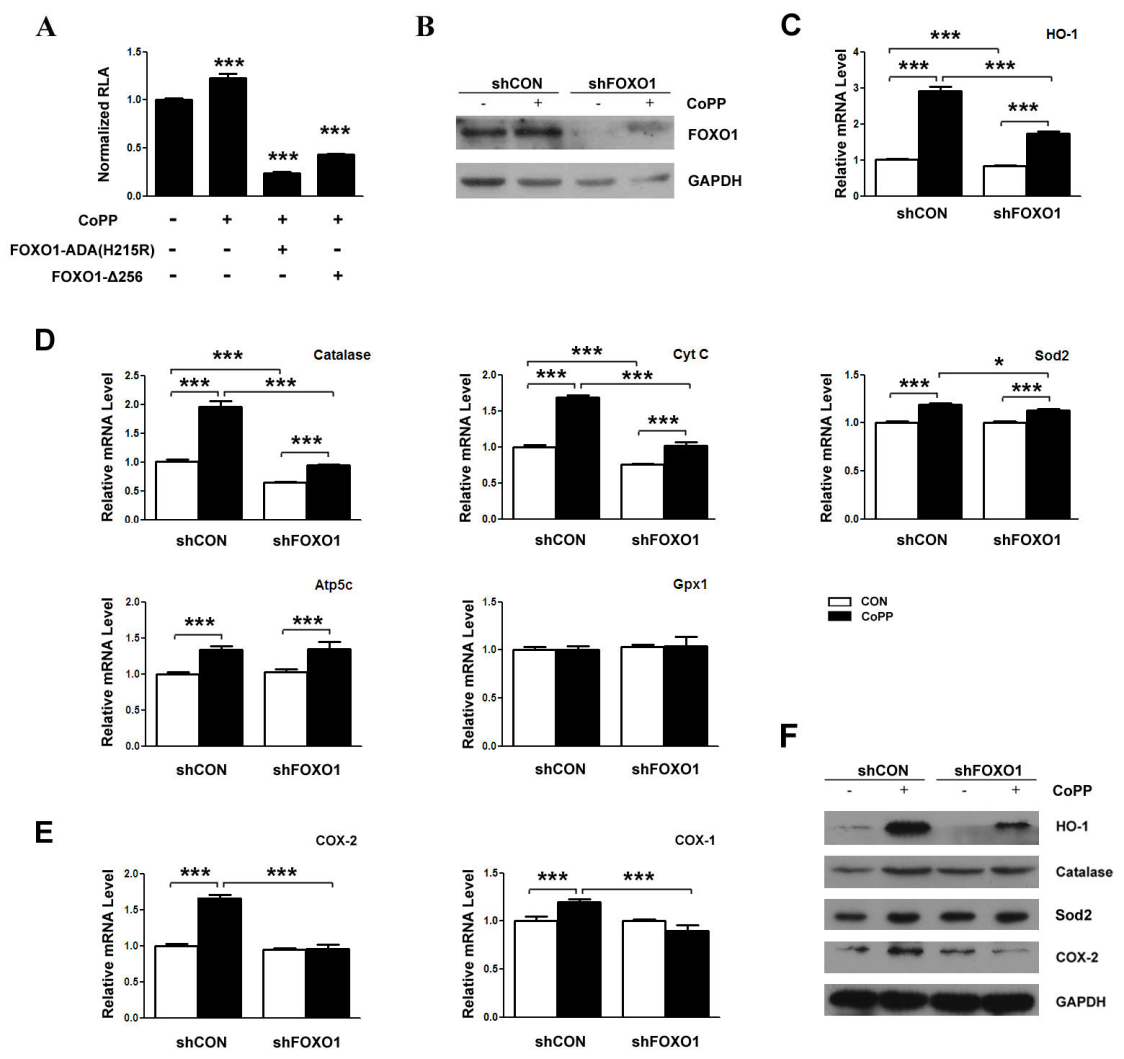
CoPP induced-expression of HO-1 and other oxidative stress-responsive genes are mediated partially by FOXO1. (A) FOXO1 mutants inhibit CoPP-induced HO-1 promoter transcription activity in HepG2 cells. HepG2 cells are co-transfected with pGL3-Basic-HO-1 promoter (200 ng) and the equivalent amounts of pcDNA3-FOXO1-ADA (H215R) ( pCMV5-FOXO1-Δ256 or control plasmids) as indicated, after which cells are treated with or without 10µM CoPP for 16 h. (B) FOXO1 expression is downregulated by shFOXO1. Primary hepatocytes are infected with shFOXO1 for 40 h, after which cells are harvested, total protein assays are carried out. (C, D, E) CoPP induced-expression of HO-1 and other oxidative stress-responsive genes are mediated partially by FOXO1. Primary hepatocytes are infected with shFOXO1 or shCON for 24 h before treatment for 16 h with and without 10µM CoPP, after which cells are harvested, total RNA assays are carried out. mRNA is quantified by qRT-polymerase chain reaction and relative amounts of mRNA are normalized to β-actin mRNA. (F) Primary hepatocytes are treated for 24 h with and without 10µM CoPP after infection with shFOXO1 or shCON for 24 h before, after which cells are harvested and total proteins assays are carried out. *p < 0.05, **p < 0.01and ***p < 0.001.

Since HO-1 is a direct target of FOXO1 and CoPP increases the expression of FOXO1, we examine whether CoPP induces HO-1 expression mediated by FOXO1. Firstly, we study the effects of CoPP and FOXO1 mutants, FOXO1-ADA(H215R) or FOXO1-Δ256, on HO-1 promoter in HepG2 cells. Our results state that CoPP increases the transcriptional activity of HO-1 promoter and both of FOXO1 mutants lessen CoPP-induced transcriptional activity of HO-1 promoter (Figure 5A). Secondly, we investigate the effect of combination of CoPP and FOXO1-shRNA on HO-1 expression in primary hepatocytes. Before testing HO-1, we confirm that the FOXO1 expression is repressed by shFOXO1 (Figure 5B). Downregulation of FOXO1 expression with shFOXO1 only slightly reduces the HO-1 mRNA expression, but obviously and incompletely weakens CoPP-induced HO-1 mRNA expression in primary hepatocytes (Figure 5C). And previous studies showed that HO-1 against oxidative stress requires an increase in Sod and catalase in experimental diabetes [29], we also detect the change of some oxidative stress-responsive genes. Our results show that CoPP enhances the mRNA expression of some oxidative stress-responsive genes including catalase, Sod2, Cyt C and Atp5c, but not Gpx1 in primary hepatocytes. And repressing FOXO1 slightly decreases the basal expression, but alleviates CoPP-induced expression of catalase and Cyt C obviously and incompletely (Figure 5D). COX-2 is the rate-limiting enzyme in prostaglandin (PG) synthesis, catalyzing the conversion of acid to PGH. Recent study indicated that COX-2 is involved in CoPP-induced HO-1 expression in human cardiac stem cells [30]. So, we also detect the effect of CoPP on the expression of COX-2 and COX-1 in primary hepatocytes. Our results reveal that CoPP enhances the expression of COX-2 and COX-1 and lower FOXO1 expression also alleviates CoPP-induced expression of COX-2 and COX-1 (Figure 5E). Similar to mRNA change, CoPP promotes the protein expression of HO-1, catalase, Sod2 and COX-2 in primary hepatocytes, which is weakened by downregulating FOXO1 expression (Figure 5F). From above results, we draw a conclusion that CoPP increases these stress-responsive genes expression, which is mediated partially by FOXO1.

### CoPP extenuates mitochondrial ROS production mediated by FOXO1

**Figure 6:**
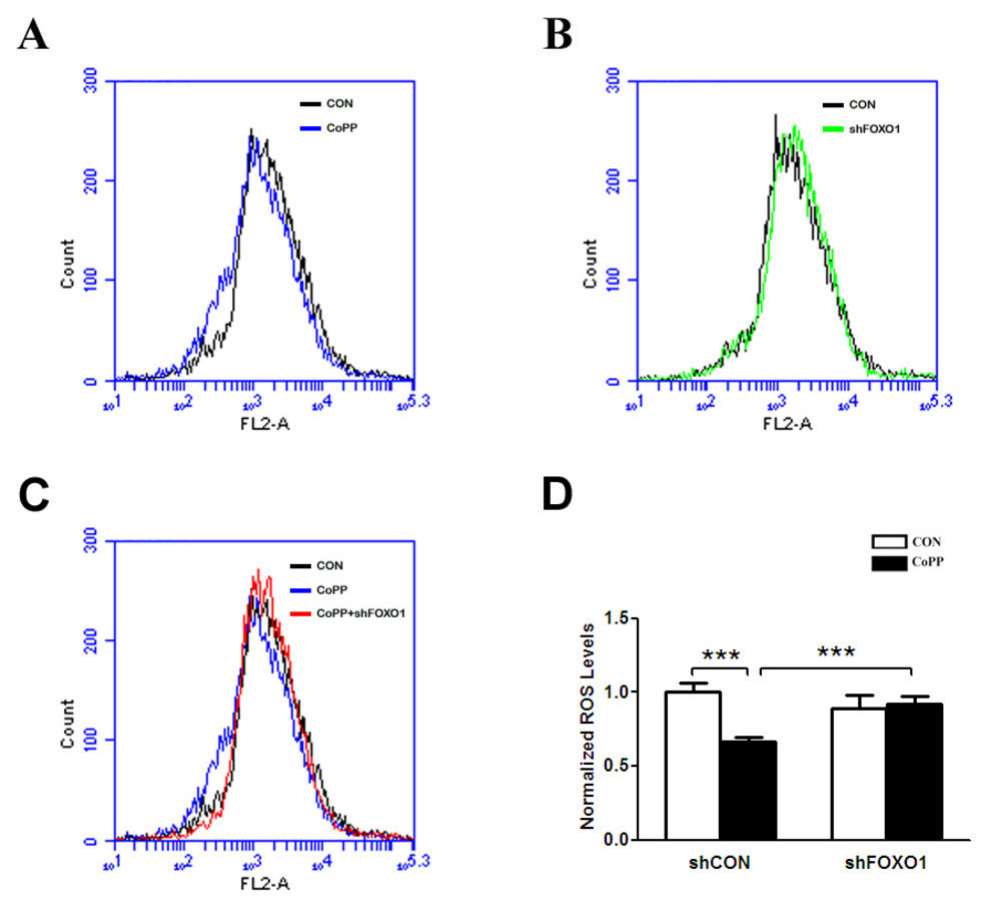
CoPP extenuates mitochondrial ROS production which is mediated by FOXO1. Primary hepatocytes are plated in six-well plates. Cells are infected by shFOXO1 virus or control virus. After 24h, cells are exposed to 10 μM CoPP or DMSO for 18h. And cells are trypsinized and harvested, then are loaded with MitoSOX Red for 10 minutes in the dark at 37°C. Next, cells are washed twice with PBS and fluorescence is measured using flow cytometry. Mitochondrial ROS levels are expressed as the mean ± s.d. intensity of cell fluorescence. ***p < 0.001 .

Since HO-1 operates on redox components through heme availability in liver mitochondria and modulates ROS production as a part of its antioxidant properties, our study indicates that CoPP affects the expression of HO-1 and other antioxidant genes. And mitochondria is an important source of ROS within most mammalian cells, we investigate the effect of CoPP on mitochondrial ROS production. The results indicate that CoPP treatment causes a decrease of mitochondrial ROS production than control (Figure 6A, 6D). Downregulation of FOXO1 expression with shFOXO1 has no obvious effect on mitochondrial ROS production compared with control (Figure 6B, 6D). However, repressed FOXO1 expression alleviates the effect of CoPP decreasing mitochondrial ROS production (Figure 6C, 6D). These data indicate that CoPP extenuates mitochondrial ROS production mediated by FOXO1. 

## Discussion

**Figure 7:**
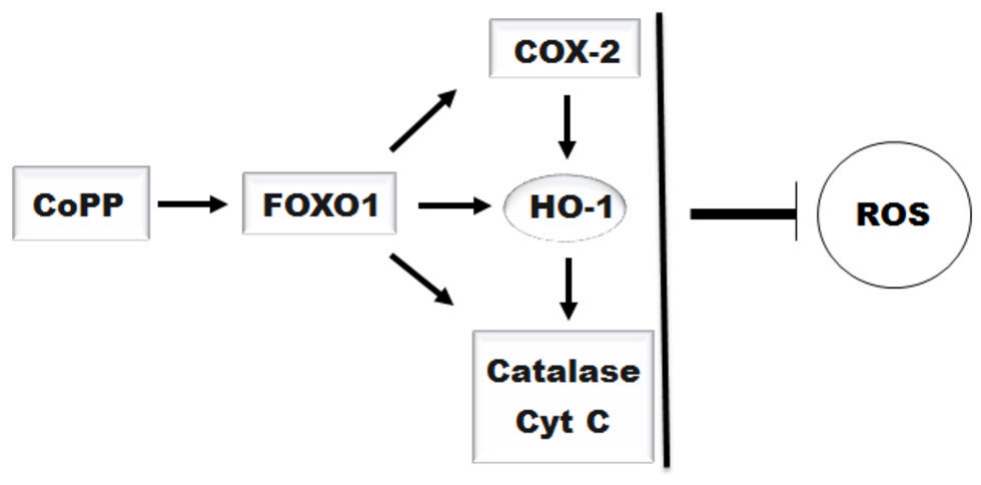
The scheme of CoPP regulates mitochondrial-derived ROS production mediated by FOXO1.

In this study, our results describe that CoPP increases the expression of FOXO1, furthermore upregulates HO-1 and other oxidative stress-responsive genes expression and decreases mitochondria-derived ROS production, which are mediated partially by FOXO1 (Figure 7).

HO-1 is an enzyme with important physiologically relevant antioxidant and cytoprotective effects by reducing the heme concentrations and producing the biliverdin and bilirubin [31,32]. Previous study indicated that FOXO1 directly regulates the expression of HO-1 by binding to the promoter of HO-1 [20]. In this study, FOXO1-ADA increases the mRNA expression of HO-1 and the transcriptional activity of HO-1 promoter, and both the mutants of FOXO1-ADA(H215R) and FOXO1-Δ256 evidently decrease the basal and FOXO1-ADA-induced transcriptional activity of HO-1 promoter. These data suggest that FOXO1 enhances the HO-1 expression through binding to and increasing the transcription activity of the HO-1 promoter. HO-1 is robustly induced in many cells by numerous stressful stimuli [10–12] and CoPP is the most efficacious metalloporphyrin inducer of HO-1 identified so far [33]. In previous studies, CoPP induces the HO-1 expression by upregulating the HO-1 promoter activity, and the pathway mediating the process includes the level changes of at least two transcription factors, namely, downregulation of Bach1 and upregulation of Nrf2 proteins by post-transcriptional mechanisms [13]. Our results show that CoPP increases the expression of HO-1 and FOXO1 in primary hepatocytes. As FOXO1 is a transcriptional factor, our results also indicate that CoPP increases the recruitment of FOXO1 to the promoter of HO-1. In addition, CoPP has no effect on FOXO1 protein stability, this is different from the previous study which has shown that CoPP affects Nrf2 and Bach1 protein stability [13]. Since HO-1 is a direct target of FOXO1 and CoPP increases the expression of FOXO1, we examine whether CoPP induces HO-1 expression mediated by FOXO1. The results suggest that FOXO1 mutants reduce HO-1 promoter activity, and that FOXO1 downregulation obviously and incompletely alleviates CoPP-induced HO-1 mRNA expression in primary hepatocytes. 

Previous studies showed that HO-1 against oxidative stress requires an increase in oxidative stress-responsive genes in experimental diabetes [29]. In this study, CoPP enhances the mRNA levels of some oxidative stress-responsive genes as catalase, Cyt C, Sod2 and Atp5c. And repressing FOXO1 expression slightly decreases the basal expression, but mitigates the CoPP-induced expression of catalase and Cyt C obviously and incompletely. It is known that these oxidative stress-responsive genes can regulate the level of ROS, and vice versa. For example, chronic overexpression of catalase eliminated excessive ROS production in diabetic cardiomyocytes and protected against diabetic cardiomyopathy caused by oxidative damage [34]. And the constitutive presence of catalase inside mitochondria is a part of the liver defense system against H_2_O_2_ [35]. In the early phase of neurotoxicity, ROS-induced cytochrome c release can be part of a cellular and mitochondrial defense mechanism against oxidative stress [36]. 

COX-2 is the rate-limiting enzyme in the synthesis of prostaglandins (PGs) from arachidonic acid. So far, two distinct COX isoforms with almost identical structure and catalytic activity have been characterized: COX-1, which is constitutive, present in nearly all cell types at a constant level; and COX-2, which is induced in response to stress but is also constitutively expressed in some tissues, implying a role for COX-2 in inﬂammation [37]. And there is a study which indicated that COX-2 can be induced by ROS [38]. Recent study indicated that COX-2 participates in CoPP-induced HO-1 expression in human cardiac stem cells [30]. Our study suggests CoPP induces COX-2 and COX-1 expression, which is attenuated by FOXO1 reduction. 

It is well known that a variety of diseases have been associated with excessive ROS. In this study, CoPP treatment causes a decrease of mitochondrial ROS production, which can be weakened by downregulating FOXO1 expression. ROS arise in the mitochondria as byproducts of respiration and oxidase activity, and cells possess intricate antioxidant defense mechanisms to counteract oxidative damage, such as, upregulation of the transcription of free radical scavenging enzymes such as Sod and catalase, by phosphorylation of the FOXOs transcription factors in response to oxidative stress [1]. Cobalt-containing compounds have been used in experimental and therapeutic applications in humans and laboratory animals including stimulating erythropoiesis in patients suffering from anemia associated with renal disease and affecting endocrine status and weight gain in experimental animals [13]. And CoPP appears to decrease oxidative stress by increasing the carnitine, citrate, deoxynucleotide, dicarboxylate, and ADP/ATP carriers content [39]. Free heme is cytotoxic, and heme clearance reduces oxidative stress and apoptosis, which is assumed to promote resolution of inflammation [40]. HO-1 operates on redox components through heme availability in liver mitochondria and modulates ROS production as a part of its antioxidant properties. This study indicates that CoPP induces HO-1 and other oxidative stress-responsive genes expression mediated partially by FOXO1, and has an important role in reducing cellular ROS level. In the future, CoPP may be a more promising therapeutic agent to upregulate some antioxidative genes.
